# Synergistic Anticancer Therapy by Ovalbumin Encapsulation‐Enabled Tandem Reactive Oxygen Species Generation

**DOI:** 10.1002/anie.202006649

**Published:** 2020-09-15

**Authors:** Shuai Jiang, Ming Xiao, Wen Sun, Daniel Crespy, Volker Mailänder, Xiaojun Peng, Jiangli Fan, Katharina Landfester

**Affiliations:** ^1^ State Key Laboratory of Fine Chemicals Dalian University of Technology 2 Linggong Road, Hi-tech Zone Dalian 116024 China; ^2^ Max Planck Institute for Polymer Research Ackermannweg 10 55128 Mainz Germany; ^3^ Department of Materials Science and Engineering School of Molecular Science and Engineering Vidyasirimedhi Institute of Science and Technology (VISTEC) Rayong 21210 Thailand; ^4^ Department of Dermatology University Clinic of the Johannes Gutenberg-University Mainz Langenbeck str. 1 55131 Mainz Germany; ^5^ Ningbo Institute of Dalian University of Technology 26 Yucai Road, Jiangbei District Ningbo 315016 China

**Keywords:** cisplatin, Fenton reactions, hypoxic, photodynamic therapy, synergistic

## Abstract

The anticancer efficacy of photodynamic therapy (PDT) is limited due to the hypoxic features of solid tumors. We report synergistic PDT/chemotherapy with integrated tandem Fenton reactions mediated by ovalbumin encapsulation for improved in vivo anticancer therapy via an enhanced reactive oxygen species (ROS) generation mechanism. O_2_^.−^ produced by the PDT is converted to H_2_O_2_ by superoxide dismutase, followed by the transformation of H_2_O_2_ to the highly toxic ^.^OH via Fenton reactions by Fe^2+^ originating from the dissolution of co‐loaded Fe_3_O_4_ nanoparticles. The PDT process further facilitates the endosomal/lysosomal escape of the active agents and enhances their intracellular delivery to the nucleus—even for drug‐resistant cells. Cisplatin generates O_2_^.−^ in the presence of nicotinamide adenine dinucleotide phosphate oxidase and thereby improves the treatment efficiency by serving as an additional O_2_^.−^ source for production of ^.^OH radicals. Improved anticancer efficiency is achieved under both hypoxic and normoxic conditions.

## Graphical abstract

Synergistic photodynamic/chemotherapy is reported with integrated tandem Fenton reactions enabled by ovalbumin co‐encapsulation. In vivo anticancer therapy is improved via an enhanced reactive oxygen species (ROS) generation mechanism.

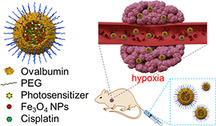

## Introduction

Cancer has been one major threat to human health for centuries.^[1]^ Various anticancer therapies including chemotherapy, radiotherapy, chemodynamic therapy, and photodynamic therapy (PDT) have been developed.^[2]^ However, treatment with a single therapy generally shows limited efficacy due to the tumor heterogeneity.^[3]^ Thus multimodal therapies that combine different treatment approaches have been broadly exploited. They show higher treatment efficiency because the active compounds with different therapeutic mechanisms could act synergistically by targeting multiple sites in the cell.^[4]^ In particular, the combination of PDT with chemotherapy presents unique advantages^[5]^ because the generated reactive oxygen species (ROS) directly damage the tumor issues^[6]^ and destroy the endosomes and lysosomes to facilitate the intracellular delivery of drugs to the targets such as nucleus, mitochondria, and microtubules.^[7]^ More importantly, ROS can reduce the drug resistance developed in chemotherapy by damaging the DNA through strong oxidation, and these damages could not be easily repaired by the drug‐resistance DNA repair enzymes.^[8]^

In spite of the promising anticancer applications of combined PDT and chemotherapy, the hypoxic feature of the solid tumors severely limits their efficacies.^[9]^ This is because the generation of ROS by traditional photosensitizers relies highly on the oxygen tension of the local microenvironment.^[10]^ Moreover, PDT can result in more severe tumor hypoxia by consuming oxygen, leading to the expression of P‐glycoprotein (P‐gp) that prevents the cellular uptake of anticancer drugs.^[11]^ Several strategies have been proposed to increase oxygen perfusion in the tumor microenvironment,^[12]^ such as the oxygen delivery systems (e.g. artificial cells^[13]^ and perfluorocarbon nanosystems^[14]^) and oxygen self‐supplement systems that are based on biological or chemical reactions (e.g. catalase, MnO_2_, and CaO_2_).^[15]^ However, these approaches only resulted in limited improvement of PDT efficiency due to the rapid consumption and slow diffusion of oxygen. Moreover, complex fabrication processes are often required.^[10a]^ Thus, developing a low oxygen‐demanding PDT system is essential for an efficient photodynamic/chemical combination therapy, which has been rarely reported.^[16]^

**Scheme 1:**
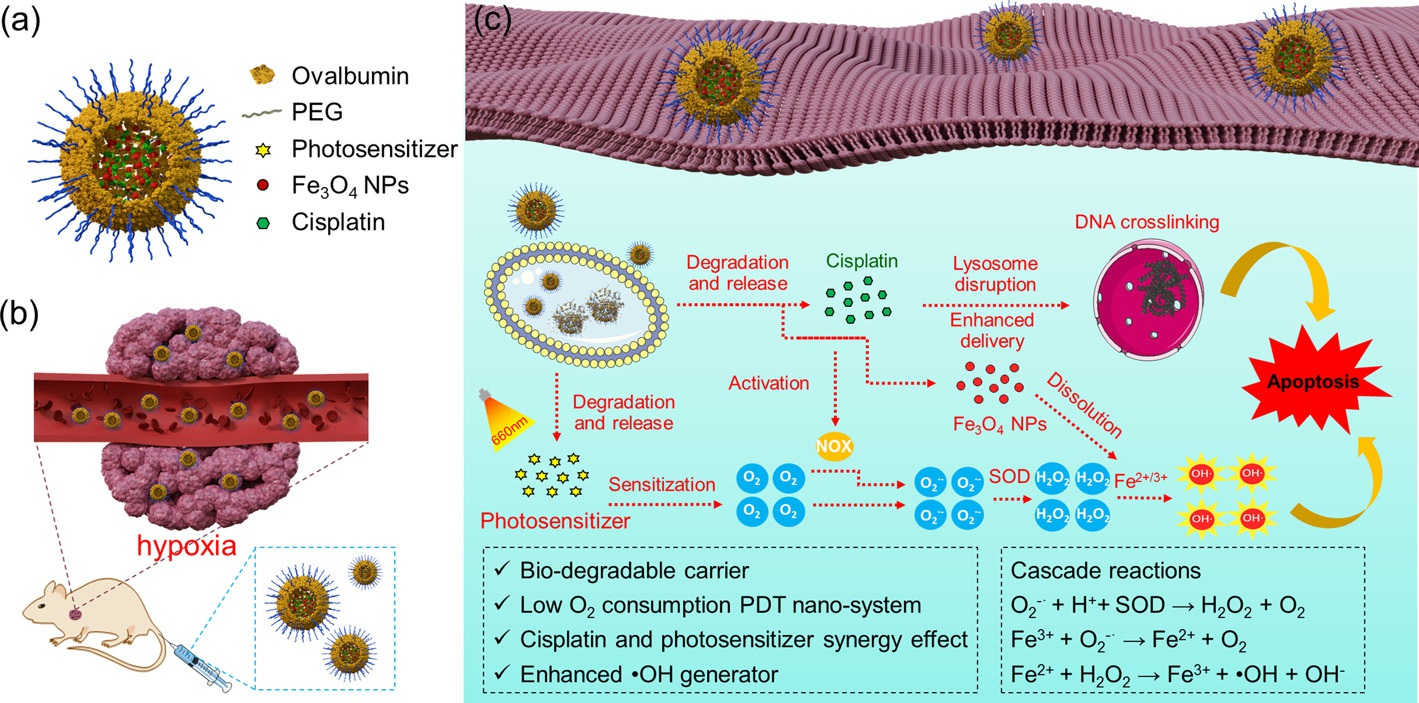
Illustration of a) the structure of the OVA‐NCs containing photosensitizer, Fe_3_O_4_ NPs, and cisplatin. b) Targeted delivery of the OVA‐NCs in the hypoxia tumor site via EPR effect. c) The mechanism of the PDT/chemotherapy combination therapy with integrated tandem Fenton reactions in the intracellular environment.

To this regard, we developed here a low O_2_ consumption type I PDT/chemotherapy combination system with integrated tandem Fenton reactions aiming to an enhanced ROS generation for improved anticancer therapy. Biodegradable ovalbumin nanocapsules (OVA‐NCs) were synthesized as nanocarriers for in vivo tumor targeted delivery of the active agents through enhanced permeability and retention (EPR) effect. The core–shell structure of the nanocapsules provides a high encapsulation efficiency for multiple payloads in the interior and payloads are selectively released upon biodegradation of the shell. Three functional components (photosensitizer, drug, and the Fenton catalyst) were co‐encapsulated in the PEGylated OVA‐NCs (Scheme 1 a). The Nile blue with S‐substitution (NBS), as the photosensitizer, is able to generate ROS via type I photoreactions under red light (656 nm) even under severe hypoxic environment (2 % O_2_, Scheme 1 b).^[17]^ The PDT produced O_2_^.−^ is partially converted to H_2_O_2_ by superoxide dismutase (SOD), and followed with the transformation of H_2_O_2_ to the highly toxic ^.^OH via Fe^2+^ mediated Fenton reactions that are enabled by the dissolution of co‐loaded Fe_3_O_4_ NPs within the cancer cells. Cisplatin is a Food and Drug Administration (FDA)‐approved anticancer drug that possesses nuclear DNA crosslinking ability, thus can restrain cell division. Moreover, cisplatin generates O_2_^.−^ in the presence of nicotinamide adenine dinucleotide phosphate oxidase (NOX). The generated O_2_^.−^ can also be partially consumed to produce toxic ^.^OH radicals, thus cisplatin can potentially improve the therapeutic efficiency by serving as an additional ROS source.^[17]^ The generated ROS from the nanocapsules can induce cell apoptosis which also benefits for endosomal/lysosomal escape of the active agents. Notably, the intracellular SOD can catalyze partial O_2_^−.^ to form hydrogen peroxide (H_2_O_2_) and O_2_. Besides, O_2_ is also released from Fenton reactions. Thus, the benefit of the system is that, at least in part, the oxygen is recyclable, which facilitates the enhancement of the anti‐hypoxia performance.^[18]^ Therefore, this multifunctional nanomedicine is designed to synergistically improve the anticancer efficiency via the combination of type I PDT and chemotherapy under both normoxic and hypoxic conditions. Moreover, this nanoencapsulation strategy provides a versatile platform for the flexible combination of different therapies for biomedical applications.

## Results and Discussion

### Encapsulation of functional PDT/chemotherapy/Fenton reaction reagents in the OVA‐NCs

We synthesized biodegradable protein nanocapsules comprising an aqueous core and a crosslinked ovalbumin shell through an interfacial polyaddition reaction in an inverse (water‐in‐oil) miniemulsion. Hydrophilic therapeutic agents were first dissolved or dispersed in an aqueous phase, which was then emulsified in cyclohexane as the continuous phase. The formed aqueous nanodroplets were stabilized with a block copolymer surfactant P(E/B‐*b*‐EO). The crosslinker 2,4‐toluene diisocyanate was then added to this miniemulsion. A polyaddition reaction between the hydroxyl and amino groups of ovalbumin and isocyanate groups of the crosslinker took place at the nanodroplet‐cyclohexane interface, resulting in water‐insoluble nanocontainers with a densely crosslinked polypeptide shell. Nanocapsules prepared in cyclohexane were then transferred to an aqueous medium with the stabilization by a PEG‐based surfactant Lutensol AT50 for enhanced colloidal stability in the blood.

For achieving a combined PDT and chemotherapy with integrated Fenton reactions, three therapeutic reagents (a photosensitizer NBS, Fe_3_O_4_ NPs for Fenton reactions, and an anticancer drug cisplatin) need to be encapsulated simultaneously in the same nanocapsule. The NBS has a good solubility in water hence it can be directly dissolved in the water phase. The Fe_3_O_4_ NPs were synthesized by a co‐precipitation approach.^[19]^ The obtained Fe_3_O_4_ NPs were capped with hydrophobic oleic acid. Therefore, these NPs were further transferred into an aqueous solution and were stabilized by poly(vinyl alcohol) (see details in the Experimental Section). The co‐encapsulation of cisplatin was challenging because of its very low solubility in water. Thus, we added an additional water‐miscible solvent (DMSO) to the aqueous phase. Cisplatin has a good solubility in DMSO at a concentration of 20 mg mL^−1^, while ovalbumin does not dissolve in DMSO. In this case, a mixture solvent of water and DMSO at a proper ratio is important to dissolve all the reagents and the ovalbumin. Finally, the optimized volume ratio of DMSO and water was determined to be 1:1, which allowed us to dissolve 5 mg cisplatin, 5 mg NBS, and 50 mg ovalbumin, and to disperse 8 mg Fe_3_O_4_ NPs in 500 μL of the water/DMSO mixture. The sample is denoted as FePtNBS@OVA. Nanocapsules containing only Fe_3_O_4_, Fe_3_O_4_ NPs plus cisplatin, Fe_3_O_4_ NPs plus NBS were prepared as control groups, which are denoted as Fe@OVA, FePt@OVA, and FeNBS@OVA, respectively. The final concentrations of payloads in the samples are listed in Table 1.

**Table 1:** Hydrodynamic diameter and *ζ*‐potential of OVA‐NCs containing various combinations of Fe_3_O_4_ NPs, cisplatin, and photosensitizer NBS.

Entry	Concentration in dispersion [mg mL^−1^]				*D*_h_^[b]^ [nm]	*ξ* ^[c]^ [mV]
	OVA‐NC^[a]^	Fe_3_O_4_	cisplatin	NBS		
Fe@OVA	1.70	0.15			312	29
FePt@OVA	2.06	0.19	0.20		316	31
FeNBS@OVA	2.13	0.19		0.20	331	35
FePtNBS@OVA	2.15	0.19	0.20	0.20	322	26

[a] Solid content of OVA‐NCs measured by freeze drying the dispersions. [b] Hydrodynamic diameter of OVA‐NCs measured in water. [c] Zeta potential measured in 1×10^−3^ m potassium chloride aqueous solution.

**Figure 1:**
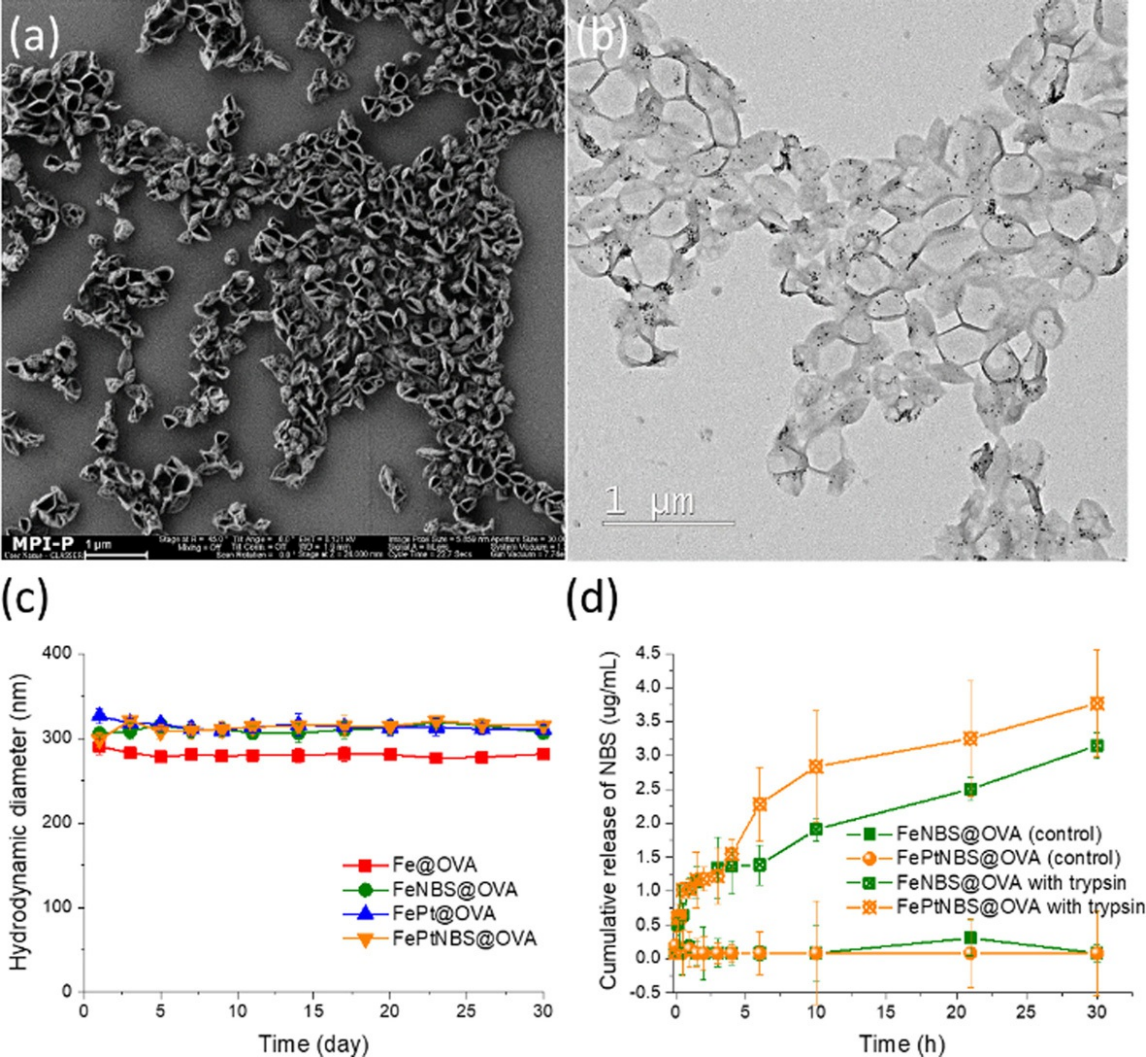
a) SEM and b) TEM images of OVA‐NCs containing Fe_3_O_4_ NPs, cisplatin, and photosensitizer NBS. c) Hydrodynamic diameter of OVA‐NCs containing various combinations of functional payloads with time up to 30 days. d) Cumulative release of NBS without and with trypsin at 37 °C. Average results of three independent release experiments are reported.

The four nanocapsule formulations with various combinations of payloads have hydrodynamic diameters around 300 nm and *ζ*‐potentials of 25–35 mV (Table 1). This positively charged surface is due to the amino groups from lysine of ovalbumin as well as the hydrolyzed unreacted isocyanate groups from the diisocyanate crosslinker after the transfer of nanocapsules to water. Core‐shell morphology of the nanocapsules (FePtNBS@OVA) was observed by using scanning (SEM) and transmission electron microscopy (TEM) (Figures 1 a,b). The nanocapsules dispersions were stable in water during a measuring time of 30 days in our study (Figure 1 c). The core–shell structure and highly crosslinked polypeptide shell are beneficial for suppressing nonspecific release of payloads. No leakage of NBS was detected after dialyzing the samples in water for 30 h (Figure 1 d). The degradability of OVA‐NCs was investigated by incubating the nanocapsules with serine protease trypsin. Continuous release of NBS from samples FeNBS@OVA and FePtNBS@OVA were observed upon incubation with trypsin (Figure 1 d), showing the preserved biodegradability of ovalbumin after the crosslinking process.

### Uptake and delivery efficiency for OVA‐NCs

Excellent ingestion efficiency of nanocarriers is a precondition for an ideal therapeutic efficacy. Cell uptake efficiency of the OVA‐NCs in MCF‐7 cells was investigated by using confocal laser scanning microscopy (CLSM). As shown in Figure S1a (Supporting Information), the red fluorescence indicates an effective internalization of NCs in the cell. Increased intensity of internalized OVA‐NCs was observed within 120 min (Figure S1b), suggesting an efficient uptake of NCs by the cancer cells.

**Figure 2:**
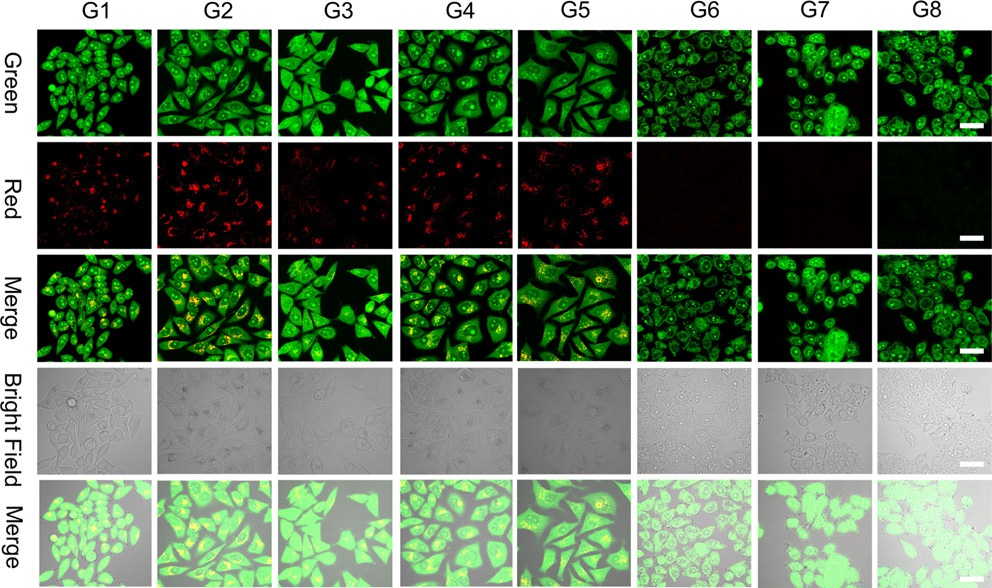
CLSM images of AO staining for assessing the lysosomal integrity. The green channel represents the fluorescence of AO monomer (*λ*_ex_=488 nm, *λ*_em_=500–550 nm), and the red channel represents the fluorescence of the dimer of AO (*λ*_ex_=561 nm, *λ*_em_=600–630 nm). Scale bar=40 μm. Key: control (G1, no capsule), light (G2), FePt@OVA (G3), FeNBS@OVA (G4), FePtNBS@OVA (G5), FeNBS@OVA+Light (G6), FePtNBS@OVA+Light (G7), FePtNBS@OVA+Light (G8) hypoxia. Light irradiation was performed by a 660 nm LED light (35 mW cm^−2^, 4 min, 8.4 J cm^−2^).

Following the endocytosis, the endosomal/lysosomal escape is the most challenging step for an efficient delivery of the payloads. The lysosomal imaging has been done to assess the ability of the OVA‐NCs for accelerating the lysosomal escape of the payloads. Acridine orange (AO) was used to characterize the lysosome membrane integrity. In the complete lysosome, the AO is in a protonated oligomeric form, showing a red fluorescence. In the cytoplasm, the AO is in a deprotonated form as a monomer that shows green fluorescence. Therefore, the intracellular distribution of AO can be used to evaluate the lysosomal membrane integrity. As shown in Figure 2, only light irradiation (G2) did not result in damage of the lysosome membrane. We also clearly observed red fluorescence in groups FePt@OVA (G3) and FePtNBS@OVA (G5), which showed that the cisplatin could not induce the damage of lysosome membrane without PDT, even though the treatments caused slight cytotoxicity inferred from the shrunk nuclei. In contrast, no red fluorescence was observed for the group FeNBS@OVA+Light (G6) and FePtNBS@OVA+Light (G7), indicating the disintegration of lysosome membranes due to the phototoxicity of PDT mediated by the NBS. Thus, the nanocapsules are able to promote intracellular drug delivery to the nuclei by facilitating the lysosomal escape via PDT. More importantly, these nanocapsules also showed an effective destruction of lysosomes under hypoxia environment (2 % O_2_) by using FePtNBS@OVA under light irradiation (G8). This result proved the applicability of the OVA‐NCs for enhanced intracellular drug delivery in the hypoxia tumor microenvironment.

Cisplatin has been well studied for inducing cell toxicity via crosslinking of DNA in the nuclei. Therefore, the accumulation of cisplatin in the nuclei determines its therapeutic efficacy. The concentration of cisplatin in the nuclei from different nanocapsule treatments has been quantified by using inductively coupled plasma mass spectrometry (ICP‐MS; Figure S2). Overall, the nanocapsules (FePt@OVA and FePtNBS@OVA) showed improved accumulation of cisplatin in the nuclei in comparison with cells treated with free drug (soluble cisplatin), which verified that the nanocarriers can indeed improve the intracellular drug delivery efficiency. After irradiation with a 660 nm light for 5 min, the Pt accumulation in nuclei further increased (Figure S2). Compared to the group treated with free cisplatin, there was an increase of 2.5 times Pt concentration in the nuclei observed for the group treated with FePtNBS@OVA+Light. Meanwhile, the light irradiation induced an increase of 1.8 times of Pt accumulation in the nuclei compared to the same group in the absence of light. Furthermore, the efficiency for nuclei accumulation has been evaluated in cisplatin resistant cell line MCF‐7/DPP. As predicted, resistant cells show negligible uptake for cisplatin due to the drug resistance. Moreover, the cells present weak accumulation of FePt@OVA and FePtNBS@OVA in nuclei after incubation. In contrast, when the FePtNBS@OVA+Light was applied, there was a significant increase of Pt accumulation in the nuclei. This result can be explained by the robust release of payloads from endosome or lysosome mediated by PDT, which ensures a considerable delivery amount of cisplatin to the nuclei. These results demonstrate that the PDT based nanocapsules can effectively enhance the intracellular delivery of cisplatin to the nuclei.

### Assessment of ROS generation in normoxic and hypoxic environments

**Figure 3:**
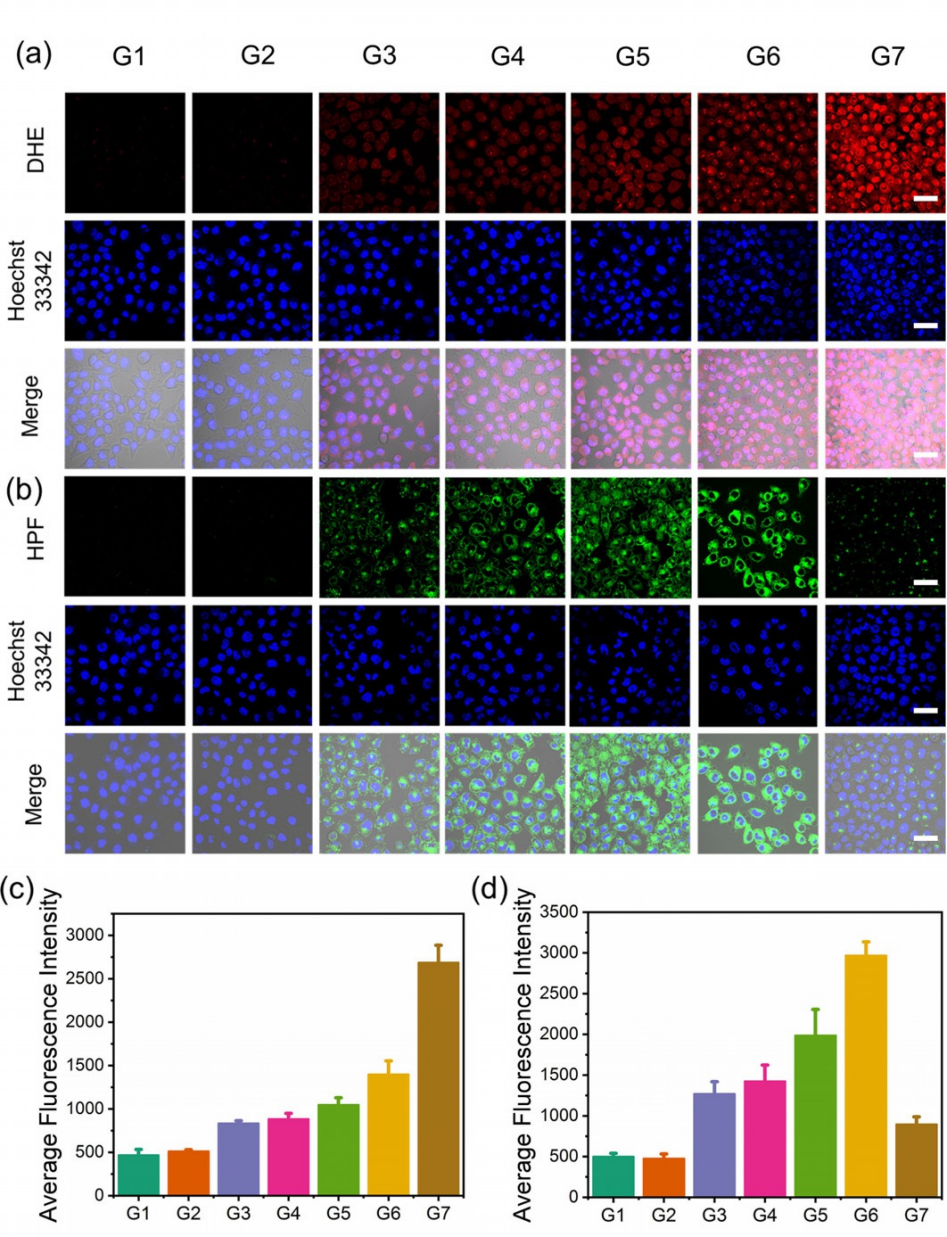
The detection of ROS generation in MCF‐7 cells under normoxia with different nanocapsule treatments by using CLSM. Key: control (G1, without nanocapsule incubation), FeNBS@OVA (G2), FePt@OVA (G3), FePtNBS@OVA (G4), FeNBS@OVA+Light (G5), FePtNBS@OVA+Light (G6), FePtNBS@OVA+DFOA+Light (G7). Light irradiation was carried out by a 660 nm LED light (35 mW cm^−2^, 4 min, 8.4 J cm^−2^). The fluorescence of Hoechst 33342 was used to stain nuclei, with excitation at 405 nm and data collection within the 420–480 nm range. Scale bar=40 μm. a) Generation of O_2_^−.^; red fluorescence was excited at 488 nm and collected at 590–610 nm. b) Generation of ^.^OH; green fluorescence was excited at 488 nm and collected at 500–550 nm. c) Average fluorescence intensity of DHE for the detection of O_2_^−.^. d) Average fluorescence intensity of HPF for the detection of ^.^OH. Data are given as the mean ± SD (*n*=3).

The photosensitizer NBS can generate O_2_^−.^ through type I photoreactions and the O_2_^−.^ can be further transformed to the highly toxic ^.^OH (Scheme 1 c). Firstly, the generation of O_2_^−.^ was demonstrated using a O_2_^−.^ fluorescence probe (DHR 123). The nanocapsules (FePtNBS@OVA) were firstly incubated with trypsin to release NBS, followed by mixing with DHR123. Red light irradiation of the solution led to significantly fluorescence enhancement, indicating the production of O_2_^−.^ under light irradiation (Figure S3). We further investigated the intracellular generation of O_2_^−.^ and ^.^OH by using a O_2_^−.^ sensor dihydroethidium (DHE) and a ^.^OH sensor hydroxyphenyl fluorescein (HPF), respectively. The MCF‐7 cells were treated with both ROS sensors and the cell images were obtained by CLSM (Figure 3). The fluorescence of both DHE and HPF was not observed for the cells incubated with FeNBS@OVA (G2). However, under 660 nm irradiation for 5 min, an obvious increase of the fluorescence intensity of both DHE and HPF was detected in the group FeNBS@OVA+Light (G5), indicating the generation of O_2_^−.^ and ^.^OH by the nanocapsules under light irradiation. The FePt@OVA (G3) and FePtNBS@OVA (G4) groups showed weak fluorescence of DHE and HPF, which indicated that cisplatin can induce the generation of O_2_^−.^ in cells. The produced O_2_^−.^ was partially converted to the ^.^OH. This is also true for the experiments under light irradiation, where a higher fluorescence intensity of both DHE and HPF were observed for the group FePtNBS@OVA+Light (G6) in comparison with that of FeNBS@OVA+Light (G5) (Figures 3 c,d). Based on this result, we further added deferoxamine (DFOA) to the cells for blocking the transformation of O_2_^−.^ to the ^.^OH. As shown in Figures 3 c,d (G6 and G7), the DFOA decreased significantly (70 % decrease) the production of ^.^OH with an increase of the fluorescence intensity from the O_2_^−.^ (FePtNBS@OVA+DFOA+Light, G7) compared to the group without DFOA (G6). These results show that (i) cisplatin alone can produce O_2_^−.^, and (ii) the NBS can produce O_2_^−.^ under light irradiation. Therefore, combined cisplatin with NBS in the nanocapsules enhanced the generation of O_2_^−.^, which can be further transformed to the highly toxic ^.^OH to achieve improved antitumor efficacy.

**Figure 4:**
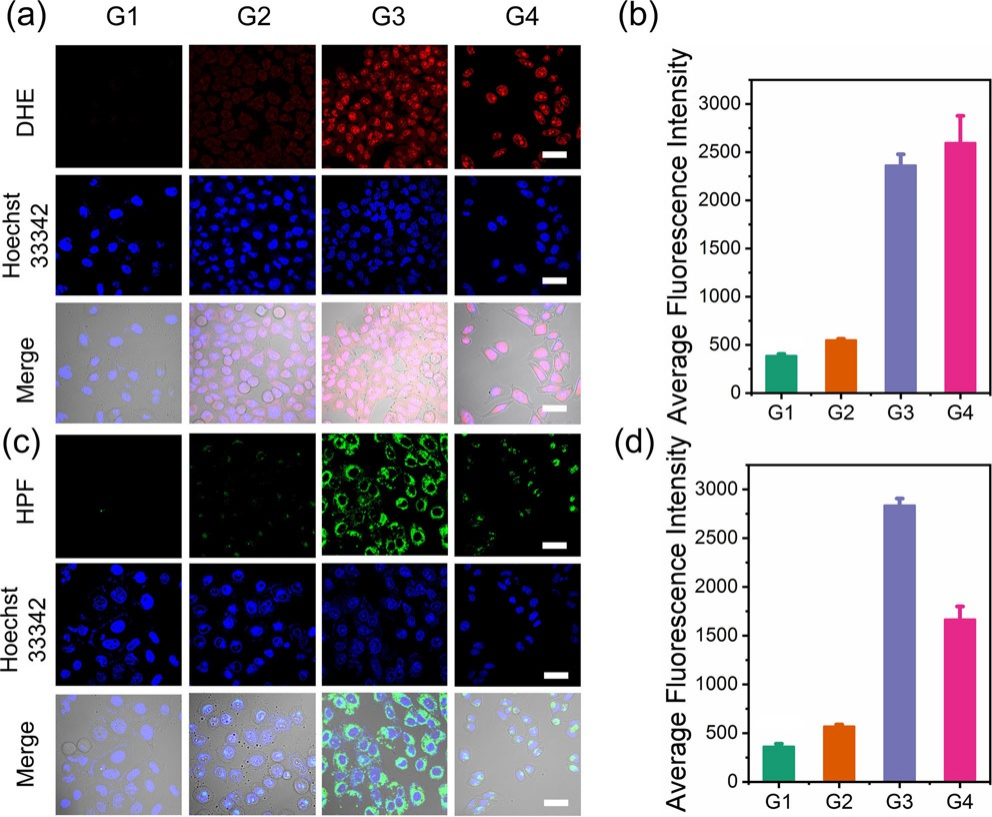
a) Detection of O_2_^−.^ (*λ*_ex_=488 nm, *λ*_em_=590–610 nm) and c) ^.^OH (*λ*_ex_=488 nm, *λ*_em_=500–550 nm) generated in MCF‐7 cells under hypoxia with different nanocapsule treatments by using CLSM. The fluorescence of Hoechst 33342 was excited at 405 nm and collected at 420–480 nm. Scale bar=40 μm. b) The quantitative analysis of fluorescence intensity of DHE for the detection of O_2_^−.^. d) The quantitative analysis of fluorescence intensity of HPF for the detection of ^.^OH. Key: control (G1), FePtNBS@OVA (G2), FePtNBS@OVA+Light (G3), FePtNBS@OVA+DFOA+Light (G4). Light irradiation was carried out by 660 nm LED light (35 mW cm^−2^, 4 min, 8.4 J cm^−2^). Data are given as the mean ± SD (*n*=3).

Hypoxia is a typical feature for the tumor microenvironment, which restricts the application of PDT and undermines the therapeutic efficacy of chemotherapy. Therefore, we assessed here the generation of ROS by the nanocapsules under hypoxic environment in order to evaluate their therapeutic efficacy. Compared to the nanocapsules FePtNBS@OVA (G4, normoxia) in Figure 3, cisplatin‐triggered generation of O_2_^−.^ can also be observed under hypoxic conditions, although the fluorescence enhancement is lower for the same sample FePtNBS@OVA (G2) in Figure 4 a. Similarly, treatment with FePtNBS@OVA+Light (G3) induced significantly enhanced generation of O_2_^−.^ (Figures 4 a,b), which proved that the nanocapsules containing NBS with light irradiation could effectively generate O_2_^−.^ in cells under hypoxic conditions. As expected, high concentrations of ^.^OH were also detected from G3 group (Figures 4 c,d). To prove the synergistic effect of PDT and Fenton reactions in hypoxic environment, we further added DFOA to the cells, because DFOA can inhibit the Fenton reactions by chelating Fe ions. As shown in Figure 4 b, the generation of O_2_^−.^ in G4 was increased slightly, due to declined transformation of O_2_^−.^. While, the presence of DFOA decreased significantly (41 % decrease) the production of ^.^OH compared to the group without DFOA (G3) (Figure 4 d). The above results showed that the synergistic effect of PDT and Fenton reactions efficiently enhanced the ROS generation even under hypoxic conditions, thus making them ideal nanosystems for in vivo anticancer treatment.

### Live/death staining and apoptosis detection

**Figure 5:**
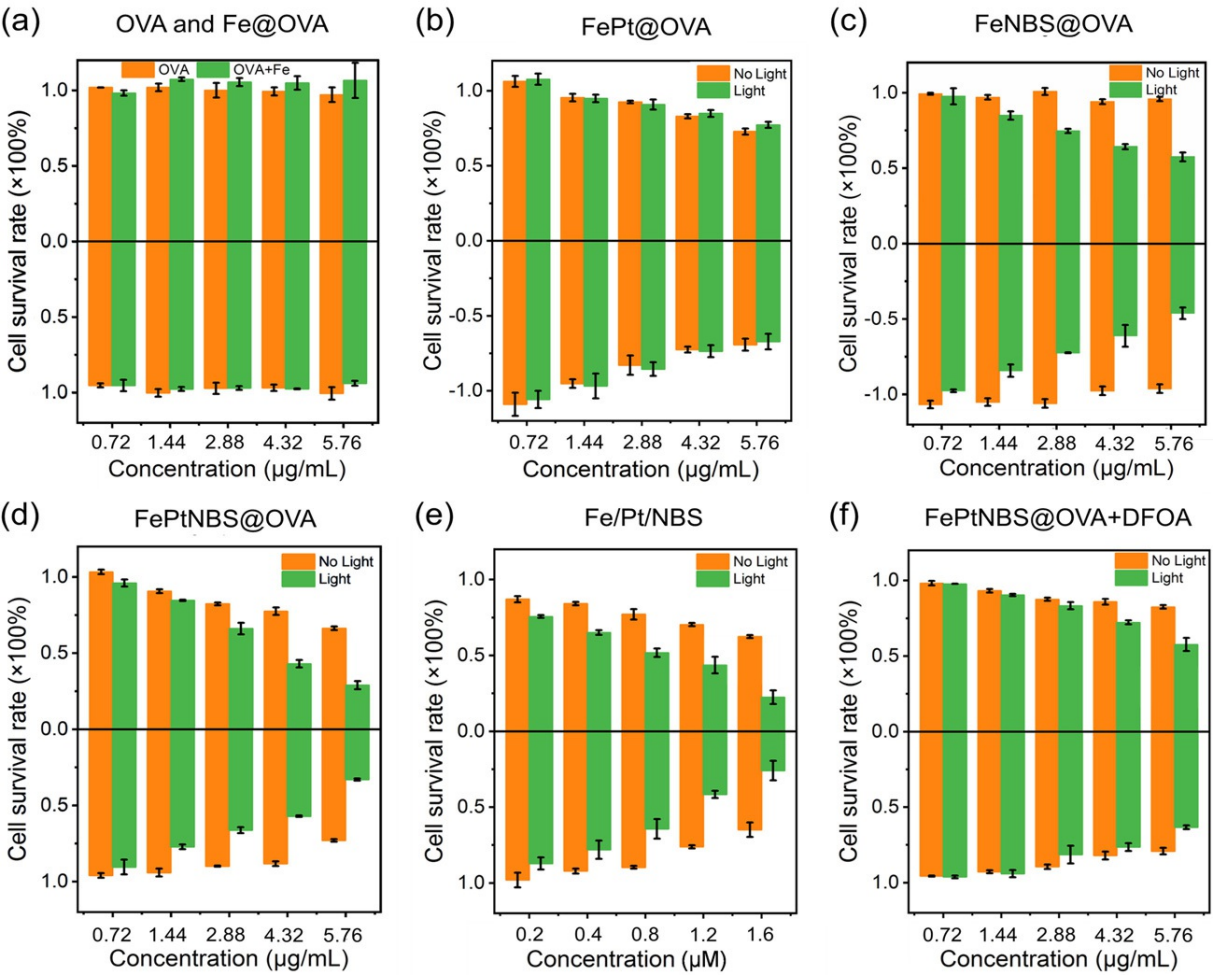
a–c) MCF‐7 (upper row) and d–f) 4T1 (lower row) cell viability after different treatments and irradiation. Data are given as the mean ± SD (*n*=5). Horizontal axis is the concentration of OVA in (a—d) and (f), and of NBS in (e). Light irradiation was carried out by 660 nm light (35 mW cm^−2^, 4 min, 8.4 J cm^−2^).

Afterwards, a Calcein‐AM/PI kit was applied to assess the therapeutic efficacy of nanocapsules in vitro. The Calcein‐AM can mark living cells with green fluorescence, and PI can track the dead cells with red fluorescence. In Figure S4d, the red fluorescence dominates the image with negligible green fluorescence when the cells were treated with the FePtNBS@OVA under light irradiation, indicating a remarkable cytotoxicity. The cytotoxicity of FePtNBS@OVA+Light (Figure 5 d) was comparable to the non‐carrier system (mixture of Fe_3_O_4_/Pt/NBS, Figure 5 e). This result showed that (i) the reagents remained their functions after the encapsulation procedure, and (ii) the nanocapsules effectively delivered the reagents into the cells and further released them in an active form based on the biodegradation of the proteins. Compared with FePt@OVA, the PDT induced by NBS caused superior therapeutic effect than the chemical toxicity induced by cisplatin (Figures S4b,c). More importantly, the combination therapy showed higher efficacy for killing cancer cells than the monotherapy (Figures S4b,c,d). Notably, the Fe@OVA did not show visible toxicity to the cells, which ensures the biosafety of the nanocapsules.

We further studied the efficiency of OVA‐NCs for inhibiting the growth of cancer cells under hypoxia conditions. The nanocapsules remained high therapeutic efficiency under hypoxic condition (Figure S4i) in comparison with the efficiency under normoxia condition (Figure S4d). The iron ions catalyze the conversion of H_2_O_2_ to ^.^OH via a Fenton reaction. To confirm this hypothesis, deferoxamine (DFOA) was added to inhibit the catalysis reaction by the iron ions in order to reduce the generation of ^.^OH. The decline of red fluorescence indicated the decreased toxicity to the cells (Figure S4j). This result validated the effectiveness of the Fenton reactions by the iron ions for an enhanced therapeutic performance.

Annexin‐FITC/PI kit was applied to study the pathways for the cytotoxicity in different treatments by using flow cytometry (Figure S5). Zone 1 represents the percentage of normal cells; zone 2 and 3 are for the cells in early and late apoptotic stage; zone 4 shows the proportion of necrotic cells. The treatment with Fe@OVA showed no detectable toxicity to the cells (Figure S5a). The toxic effect mediated by sole chemotherapy and sole PDT induced late apoptosis for 36.4 % (Figure S5b) and 75.0 % (Figure S5d) of the cells, respectively. Remarkably, up to 92.1 % late cell apoptosis was achieved in the FePtNBS@OVA+Light group (Figure S5e), demonstrating that the combination of PDT with chemotherapy induced an enhanced toxicity towards cancer cells. Moreover, when DFOA was added, the toxicity of the combined therapy declined significantly (Figure S5c), which indicated that the catalysis of Fe_3_O_4_ contributed to the excellent anticancer effect. These results indicated that the design of the nanocapsules achieved promising therapeutic effect based on combined type I PDT and chemotherapy with integrated tandem Fenton reactions.

### Toxicity assessment

Furthermore, MTT (Thiazolyl Blue Tetrazolium Bromide) was used to assess the toxicity with different treatments on MCF‐7 and 4T1 cells (Figure 5). The group for OVA and Fe@OVA showed ≈100 % viability at the concentration of 5.76 μg mL^−1^ (Figure 5 a). The group FePt@OVA resulted in limited therapeutic efficiency. About 25 % cells were killed when the concentration of capsules was 5.76 μg mL^−1^ (Figure 5 b). The treatment based on FeNBS@OVA without light showed negligible toxicity, while the irradiation at 35 mW cm^−2^ for 4 min induced 40 % cell death (Figure 5 c). Further introducing cisplatin to the PDT system led to a total cell death of 80 % (Figure 5 d). Moreover, the nanocapsules demonstrated comparable therapeutic effects compared to the mixture dose without carrier (Figure 5 e). Addition of DFOA also declined the toxicity to MCF‐7 and 4T1 cells (Figure 5 f), which indicated that the transformation from O_2_^−.^ to ^.^OH is vital for enhanced therapeutic efficiency. These results showed that the co‐delivery system possesses excellent internalization efficiency and enhanced cytotoxicity.

**Figure 6:**
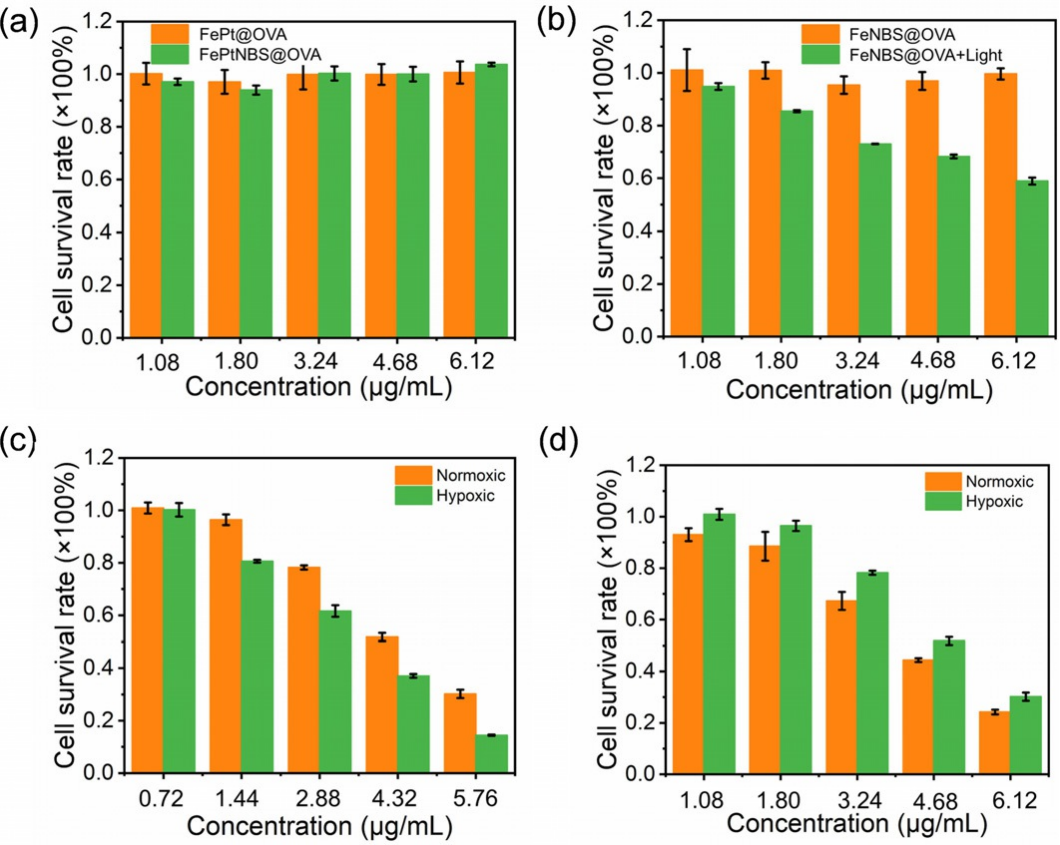
a) MTT test in cisplatin‐resistant cells (MCF‐7/DDP) from the treatment with FePt@OVA and FePtNBS@OVA. b) MTT test in MCF‐7/DDP from the treatment with FeNBS@OVA and FeNBS@OVA+Light. c) MTT test in MCF‐7 from the treatment with FePtNBS@OVA+Light in normoxic or hypoxic environment. d) MTT in MCF‐7/DDP from the treatment with FePtNBS@OVA+Light in normoxic or hypoxic environments. Horizontal axis is the concentration of OVA in all images in this Figure. Light irradiation was carried out by 660 nm LED light (35 mW cm^−2^, 4 min, 8.4 J cm^−2^).

Moreover, we evaluated the therapeutic effect of nanocapsules in cisplatin resistant cells (MCF‐7/DDP). The treatment with FePt@OVA and FePtNBS@OVA showed no toxic effect to the resistant cells (Figure 6 a). Without light irradiation, the treatment with FeNBS@OVA did not present detectable toxicity to the cells (Figure 6 b). However, when the light illumination was applied, an increased cytotoxicity was observed as the nanocapsules concentration increased. The PDT/chemotherapy (FePtNBS@OVA) showed the highest toxicity (IC_50_ ≈4 μg mL^−1^) for the resistant cells (Figure 6 d). The efficacy of the combination therapy was investigated under hypoxic and normoxic conditions. The nanocapsules showed a concentration‐dependent cell killing ability in both environments (Figure 6 c). Importantly, the significant toxicity to the resistant cells was also observed under both oxygen conditions (Figure 6 d). The IC_50_ in normoxic environment is about 5.5 μg mL^−1^, and 6.5 μg mL^−1^ in hypoxic environment, which means that FePtNBS@OVA can induce significant toxicity towards the resistant cells in both normoxic and hypoxic environments.

### In vivo assessment of therapeutic efficacy

**Figure 7:**
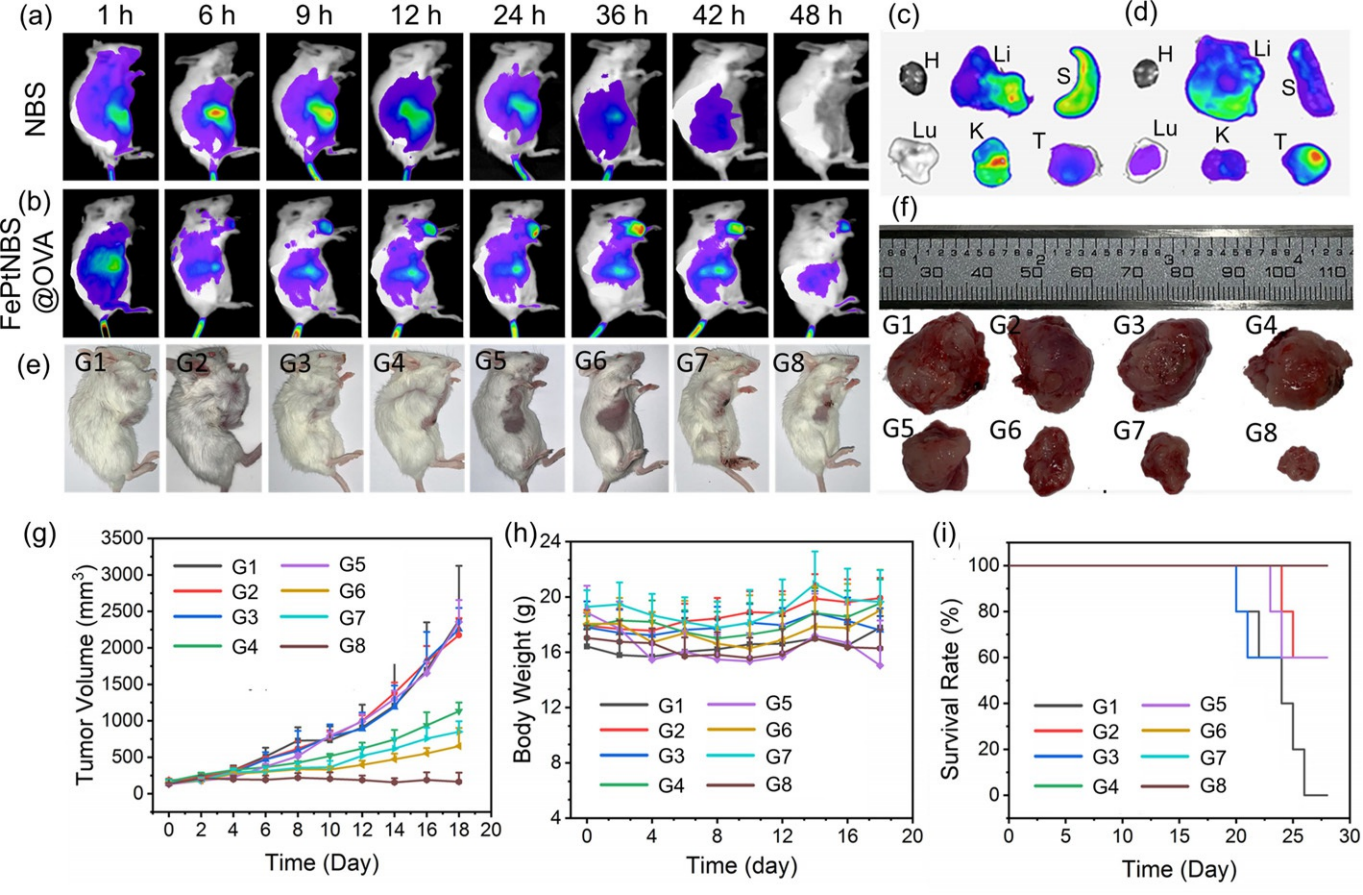
In vivo fluorescence imaging within 48 h after intravenous injection of a) NBS (100 μL, 0.2 mg mL^−1^) and b) FePtNBS@OVA (100 μL, 0.2 mg mL^−1^, referring to the concentration of NBS in the nanocapsule) from the tail view. c,d) Ex vivo fluorescence imaging of important organs of NBS‐ (c) and FePtNBS@OVA‐ (d) treated mouse, when the accumulation reached the highest point at 36 h. Key: heart (H), liver (Li), spleen (S), lung (Lu), kidney (K), tumor (T). e) Photographs of the tumor‐bearing mice at the 28th day after treatment with different formulations. Key: control (G1), Fe@OVA (G2), FePtNBS+Light (G3), FePt@OVA (G4), FeNBS@OVA (G5), FeNBS@OVA+Light (G6), FePtNBS@OVA (G7), FePtNBS@OVA+Light (G8). Light irradiation was carried out by a 660 nm xenon lamp (150 mW cm^−2^, 20 min, 180 J cm^−2^). f) Photographs of the isolated tumor tissues from tumor‐bearing mice after different treatments. g) Time‐dependent tumor growth profiles during treatment. h) Changes in mouse body weight over time after treatment. i) Survival rate curve of 4T1 tumor‐bearing mice after various treatments. Five mice were chosen in each group for treatment experiments.

Tumor‐bearing mice model was applied to assess the therapeutic efficiency of the nanoformulations in vivo. Considering that it is difficult to cultivate tumors in normal mice by using human breast cancer cells (MCF‐7), we selected 4T1, mice breast cancer cells, to establish the tumor‐bearing mice model. When the tumor volume reached approximately 100 mm^3^, we started the treatment by using the nanocapsules. In fact, the internal part of solid tumors has a hypoxic microenvironment, especially in the areas away from the blood vessels (>70 μm; Figure S6).[^6a^, ^20^] As shown in Figure 7 b, the FePtNBS@OVA nanocapsules presented preferential accumulation in the tumor site compared to the free NBS (Figure 7 a) due to the EPR effect. The nanocapsule accumulation reached maximum in the tumor at 36 h, while the free NBS was cleared from the blood circulation quickly. A weak accumulation of free NBS in tumor site was observed (Figure 7 a). In comparison, there was still considerable retention of nanocapsules in the tumor until 48 h, which guaranteed a sufficient time for the degradation of nanocapsules and hence an effective release of functional payloads. The results suggested that the nanocapsule carriers effectively extended the blood circulation time of therapeutic agents and increased accumulation in the tumor site. The fluorescence images of isolated tissues for the two groups further validated the preferable enrichment of nanocapsules in the tumor (Figures 7 c,d). The photographs of the tumor‐bearing mice 28 days after the treatment with different formulations are shown in Figure 7 e. The evolution of the tumor volume was monitored during the treatment (Figure 7 g). The loading of cisplatin led to a decrease of the tumor volume (G4, G7). The light irradiation effectively improved the therapeutic efficacy of PDT by comparing the results of FeNBS@OVA without or with light irradiation (G5, G6). Remarkably, the combined PDT and chemotherapy completely inhibited the growth of tumor (FePtNBS@OVA+Light, G8). Additionally, the combination of free agents (G3) was not able to inhibit the tumor growth, because small molecules can be easily cleared from the blood circulation, resulting in low tumor accumulation. The images of isolated tumors in Figure 7 f also demonstrated that the treatment in G8 can effectively inhibit the tumor growth.

The body weight changes of the mice were recorded over the time range of treatment. No significant change of body weight was observed for all the groups, indicating the negligible systemic toxicity of the nanocapsules (Figure 7 h). The survival rate of mice over time was recorded. The groups received the combined PDT and chemotherapy prolonged lifespan of tumor‐bearing mice (Figure 7 i). All mice survived during a one‐month monitoring period. The toxicity of different treatments was also estimated via histological analysis of the major organs (heart, liver, spleen, lung, and kidney) of sacrificed mice after treatments (Figure S7). These organs in all groups did not show any pathological tissue damages or abnormality, verifying the high biosafety of the OVA‐NCs.

## Conclusion

A low O_2_ consumption PDT/chemotherapy system was developed with integrated cascade Fenton reactions for combined anticancer treatment by using biodegradable ovalbumin nanocapsules as the nanocarriers. The core–shell nanocapsules showed a high encapsulation efficiency for the co‐encapsulation of multiple functional payloads (photosensitizer, cisplatin and Fe_3_O_4_ NPs) and their selective release based on the biodegradation of the nanocapsule. The photosensitizer NBS was able to generate ROS via type I photoreactions under red light (656 nm) even under a severe hypoxic environment (2 % O_2_), followed by the transformation of O_2_^−.^ to highly toxic ^.^OH radicals via Fe^2+^‐involved Fenton reactions that are enabled by the dissolution of loaded Fe_3_O_4_ NPs within the cancer cells. PDT also facilitated the endosomal/lysosome escape of the payloads. Cisplatin generates O_2_^.−^ in the presence of NOX and SOD, therefore it improves the PDT efficiency as an additional O_2_^.−^ source for the production of toxic ^.^OH radicals. The developed nanomedicine showed an improved anticancer efficiency via combined type I PDT and chemotherapy with subsequent Fenton reactions under both normoxic and hypoxic conditions. Moreover, this nanoencapsulation strategy provides a versatile platform for the flexible combination of different therapies for biomedical applications.

## Conflict of interest

The authors declare no conflict of interest.

## Supporting information

As a service to our authors and readers, this journal provides supporting information supplied by the authors. Such materials are peer reviewed and may be re‐organized for online delivery, but are not copy‐edited or typeset. Technical support issues arising from supporting information (other than missing files) should be addressed to the authors.

Supplementary
