# Supplementary material for: Synergistic Anticancer Therapy by Ovalbumin Encapsulation‐Enabled Tandem Reactive Oxygen Species Generation
Source: Angew Chem Int Ed Engl. 2020 Sep 15;59(45):20008–16. doi: 10.1002/anie.202006649 (PMC7693068; doi:10.1002/anie.202006649)
Supplement: Supplementary file 1 — Supplementary [file ANIE-59-20008-s001.pdf]

Angewandte Chemie
Eine Zeitschrift der Gesellschaft Deutscher Chemiker

Supporting Information

Synergistic Anticancer Therapy by Ovalbumin Encapsulation-Enabled Tandem Reactive Oxygen Species Generation

Shuai Jiang[+], Ming Xiao[+], Wen Sun, Daniel Crespy, Volker Mailänder, Xiaojun Peng, Jiangli Fan,* and Katharina Landfester**

anie_202006649_sm_miscellaneous_information.pdf

Contents

1. Experimental Section

1.1 Materials for Nanocapsule Preparation

1.2 Reagents and Instruments for Biological Experiments

1.3 Synthesis of Photosensitizer Nile blue with S-substitution (NBS)

1.4 Synthesis of Water-Dispersible Iron Oxide Nanoparticles (Fe_3O_4 NPs)

1.5 Synthesis of Ovalbumin Nanocapsules

1.6 Biodegradability of Nanocapsules and Release of Payloads

1.7 Characterizations of Nanocapsules

1.8 Cell Incubation

1.9 Cell Imaging Experiments

1.10 Assessment for Intranuclear Pt Content

1.11 Cytotoxicity Test

1.12 Lysosome Disruption Assay

1.13 Intracellular Superoxide Anion Free Radical Detection

1.14 Intracellular Hydroxyl Radical Detection

1.15 Live-Dead Cell Staining

1.16 Apoptosis Experiment

1.17 *In vivo* Fluorescence imaging

1.18 *In vivo* Therapeutic Efficacy

2. Results

3. Animal Experimental Ethical Inspection Form

1. Experimental Section

1.1 Materials for Nanocapsule Preparation

Albumin from chicken egg white (lyophilized powder, ≥98%, Sigma Aldrich), sodium chloride (NaCl, ≥99.0%, Sigma Aldrich), dimethyl sulfoxide (DMSO, <50 ppm water content, Acros Organics), *cis*-diamineplatinum(II) dichloride (cisplatin, ≥99.9% trace metals basis, Sigma Aldrich), cyclohexane (HPLC grade, VWR), 2,4-toluene diisocyanate (TDI, 99%, Sigma Aldrich), and sodium dodecyl sulfate (SDS, 99%, Alfa Aesar) were used as received. The surfactant poly((ethylene-*co*-butylene)-*block*-(ethylene oxide)), P((E/B)-*b*-EO), consisting of a poly(ethylene-*co*-butylene) block (NMR: M_n = 3900 g mol^{-1}) and a poly(ethylene oxide) block (NMR: M_n = 2700 g mol^{-1}) was synthesized according to a reported procedure.[1] Milli-Q water was used in all experiments.

1.2 Reagents and Instruments for Biological Experiments

General chemicals including 3-(4,5-dimethylthiazol-2-yl)-2,5-diphenyltetrazolium bromide (MTT) and 1,3-diphenylisobenzofuran (DPBF) were purchased from Energy Chemical Co. 3'-(4-hydroxyphenyl)luciferin (HPF, Molecular Probes Invitrogen) was purchased from Sigma-Aldrich. The dihydroethyl ingot (DHE) and AV-FITC/PI apoptosis detection kits were purchased from Beyotime biotechnology Co., Ltd. All other solvents and reagents used in this study were of analytical grade. The data for MTT is obtained from Thermo Scientific™ Varioskan™ LUX multifunctional microplate reader. Confocal laser scanning microscopy (CLSM) images were performed on an Olympus FV1000-IX81 confocal laser scanning microscope. Fluorescence imaging of small animals was performed by the NightOWL II LB983 imaging system. The Pt content was determined by PerkinElmer NexION 2000 ICP-MS.

1.3 Synthesis of Photosentisizer NBS

The synthesis of photosentisizer NBS was reported in a previous procedure (compound b).[2]

1.4 Synthesis of Water-Dispersible Iron Oxide Nanoparticles (Fe_3O_4 NPs)

Oleic acid capped Fe_3O_4 NPs were synthesized by a standard co-precipitation protocol.[3] Iron(III) chloride hexahydrate (24.36 g, 90 mmol) and iron(II) chloride tetrahydrate (12.01 g, 60 mmol) were dissolved in 100 mL deionized water. 40 mL of a 28% ammonia solution was added dropwise under mechanical stirring at 700 rpm and oleic acid (4.00 g, 14 mmol) was subsequently added. The reaction mixture was heated to 70 °C for 1 h under stirring at 500 rpm and afterwards at 110 °C for 2 h. After precipitation of Fe_3O_4 NPs capped with oleic acid, the precipitate was rinsed five times with deionized water and dried in an oven at 65 °C overnight. The Fe_3O_4 NPs were transferred into water solutions for encapsulation in nanocapsules according to our previous work.[4]

1.5 Synthesis of Ovalbumin Nanocapsules

First, 50 mg ovalbumin and 7.2 mg NaCl were dissolved in 500 μL water at a stirring rate of 200 rpm. For the encapsulation of therapeutic agents, calculated amounts of Fe_3O_4 NPs, cisplatin, and/or NBS were added in the aqueous phase at this step. Next, 35.8 mg surfactant P((E/B)-*b*-EO) was dissolved in 7.5 g cyclohexane. The mixture was poured to the aqueous phase under stirring at 500 rpm. The pre-emulsion was homogenized by ultrasonication for 180 s (30 s ultrasonication, 10 s pause) with ice cooling at 70% amplitude using a Branson 450W sonifier and a 1/2’ tip. Separately, 10.7 mg P((E/B)-*b*-EO) was dissolved in 5 g cyclohexane and 2 mg TDI was added to the solution. This mixture was added dropwise to the obtained miniemulsion for 5 min and the reaction was allowed to proceed for 24 h at 25 °C. Afterwards, excessive

surfactant was removed from the dispersion by repetitive centrifugation and replacement of the supernatant with fresh cyclohexane.

Next, the obtained nanocapsules (in cyclohexane) were transferred to an aqueous medium. 600 μL dispersion from cyclohexane was added dropwise to 5 mL 0.1 wt% SDS aqueous solution placed in an ultrasound bath. Subsequently, the sample was stirred with an open cap overnight to evaporate the cyclohexane. Excess SDS was removed *via* four centrifugation steps by replacing the supernatant with water.

For loading the nanocapsules with therapeutic agents, 250 μL Fe_3O_4 NPs dispersion (30 mg mL^{-1} in water) and 250 μL DMSO solution containing 20 mg cisplatin or/and 20 mg NBS were used and the amount of water was reduced accordingly to maintain 500 μL as the total volume for the water phase.

1.6 Biodegradability of Nanocapsules and Release of Payloads

Degradation of ovalbumin nanocapsules was studied by incubating the nanocapsule dispersions with serine protease trypsin. Specifically, 1 mL of dispersions with a solid content of 2wt% were incubated with 20 mg trypsin (gibco) at 37 °C. The mixture solutions were placed in dialysis tubes with molecular weight cut-off of 14 kDa. The dialysis tubes were then immersed in 20 mL milli-Q water and incubated at 37 °C in a shaking culture incubator. During the release experiment, 1 mL of dialysis medium was taken at given intervals and equal volume of water was added to keep the volume constant. Nanocapsules was treated in the same way in the absence of trypsin as control groups. The release of NBS in dialysis medium was quantified by measuring its absorbance at λ = 660 nm by using an Infinite M1000 plate reader (Tecan, Austria). A calibration curve of absorption against concentration of NBS is provided in the *Supporting Information*.

1.7 Characterizations of Nanocapsules

Hydrodynamic diameter of nanocapsules in water was measured by using dynamic light scattering (DLS, Malvern S90) at 25 °C at a fixed scattering angle of 90°. Colloidal stability of nanocapsules containing various combinations of Fe_3O_4 NPs, cisplatin, and NBS was studied by DLS for 30 days. The morphology of nanocapsules was examined with a Gemini 1530 (Carl Zeiss AG, Oberkochem, Germany) scanning electron microscope (SEM) operating at 0.35 kV and a JEOL 1400 (Jeol Ltd, Tokyo, Japan) transmission electron microscope (TEM) operating at an accelerating voltage of 120 kV. SEM and TEM samples of nanocapsules were prepared by casting the diluted dispersions from cyclohexane on silicon wafers and carbon layer-coated copper grids, respectively. Zeta potential of nanocapsules was measured in $1 \cdot 10^{-3}$ M potassium chloride solution at 25 °C using a Malvern Zeta sizer (Malvern Instruments, UK).

1.8 Cell Incubation

MCF-7, 4T1, and MCF-7/DDP cells were cultured in DMEM medium (DMEM, Invitrogen) containing 10% fetal bovine serum and 1% double antibody (cyanin mixture) and incubated at 37 ºC under 5% CO_2. When the hypoxic experiments are carried out, the concentration of O_2 is controlled below 2%.

The details for hypoxic experiments: fresh medium was bubbled with nitrogen for 30 min to obtain hypoxic medium. Then all the cells for hypoxic experiments will incubate in this medium. Before the cells are used for experiments, the incubators are kept in incubator chamber (MIC-101, Billups-Rothenberg) at 37 °C in a humidified, 2% O_2, 5% CO_2, and 93% N_2 atmosphere for more than 8 h. Meanwhile, an oxygen detector (Nuvair, O_2 Qucikstick) was used to real time monitor the oxygen content (≈ 2% O_2) in the chamber.

1.9 Cell Imaging Experiments

MCF-7 cells were planted onto 35 mm confocal dishes and incubated at 37 °C under 5% CO_2 for 24 h. After obtained 80% cell density, the cells were washed by PBS for

three times, then nanocapsule dispersion (2 μg mL^{-1}) was added. The images were collected at specific times. The red fluorescence was excited at 635 nm, and collected at 650-700 nm. The fluorescence of Hoechst 33342 was excited at 405 nm and collected on 420-480 nm.

1.10 Assessment for Intranuclear Pt Content

MCF-7 and MCF-7/DDP cells (approximately 2×10^5 cells/mL) were seeded into 6-well plates. After 24 h of culture, the cells were washed by PBS for three times. The drug concentrations for different administrations were calculated by using the concentration of Pt (1.5 μM). After incubating cells for 2 h, the irradiation (660 nm LED light, 35 mW/cm^2, 5 min, 10.5 J/cm^2) was elicited. Then, the cells were incubated for another 1 h. After that, the cells were collected into tubes. Nuclear extracts were obtained using a Nuclear/Cytosol Fractionation Kit (BioVision Research). The nuclear extraction is a standard method that is widely used.[5-7] Finally, the Pt contents were determined by ICP-MS (PerkinElmer NexION 2000 ICP-MS).

1.11 Cytotoxicity Test

Cell viability was assessed on formazan crystals by MTT (3-(4,5)-dimethylthiazole(-2-yl)-3,5-diphenyltetrazolium) using mitochondrial dehydrogenase. MCF-7, 4T1, and MCF-7/DDP cells were seeded at a density of 1×10^5 cells mL^{-1} in 96-well microplates and cultured in 150 μL of medium containing 10% FBS. After the cells were attached for 24 h, the plates were washed with 150 μL/well of PBS. The cells were then incubated with DMEM containing different dosage forms with different concentrations. The experimental group without irradiation was returned to the incubator for further 24 h. The group that required irradiation was incubated with these agents for 2 h, followed by irradiation with a 660 nm LED light (35 mW/cm^2, 4 min, 8.4 J/cm^2). After that, cells were further incubated in the incubator for 24 h.

The details for hypoxic experiments: fresh medium was bubbled with nitrogen for 30 min to obtain hypoxic medium. Then all the cells for hypoxic experiments were incubated in this medium. Before the cells are used for experiments, the incubators were kept in an incubator chamber.

For viability test, 10 μL MTT (5 mg mL^{-1}) prepared in PBS was added to each well, and the plate was incubated at 37 °C for 4 h in a 5% CO_2 humidified incubator. The medium was then carefully removed and the purple crystals were dissolved in 150 μL of DMSO. The absorbance at 570 nm was measured on a microplate reader (Thermo Fisher Scientific). Cell viability = ($OD_{experimental\ group}$ - $OD_{blank\ control}$) / ($OD_{negative\ control}$ - $OD_{blank\ control}$) × 100%, wherein the negative and blank controls were the unmedicated group and the blank medium group, respectively.

1.12 Lysosome Disruption Assay

MCF-7 cells were placed onto 35 mm confocal dishes and incubated at 37 °C under 5% CO_2 for 24 h. Then cells were under different treatments: G1: Control (no capsule); G2: Light; G3: FePt@OVA; G4: FeNBS@OVA; G5: FePtNBS@OVA; G6: FeNBS@OVA+Light; G7: FePtNBS@OVA+Light; G8: FePtNBS@OVA+Light, Hypoxia. The concentration of nanocapcules is 4 μg mL^{-1}, and the concentration was measured as the content of ovalbumin in the dosage form. The group that required irradiation was incubated with these agents for 2 h, followed by irradiation with a 660 nm LED light (35 mW/cm^2, 4 min, 8.4 J/cm^2). Before imaging experiments, all cells were stained with AO (5 μM) for another 0.5 h. The excitation wavelength of AO was 488 nm, and capture emission region was 515-545 nm for green channel and 610-640 nm for red channel.

1.13 Intracellular Superoxide Anion Free Radical Detection

MCF-7 cells (approximately 2×10^5 cells mL^{-1}) were seeded into 35 mm diameter confocal dishes. Then cells were under different treatments: G1: Control (without

nanocapsule incubation); G2: FeNBS@OVA; G3: FePt@OVA; G4: FePtNBS@OVA; G5: FeNBS@OVA+Light; G6: FePtNBS@OVA+Light; G7: FePtNBS@OVA+DFOA+Light. Light irradiation was carried out by 660 nm LED light (35 mW/cm^2, 4 min, 8.4 J/cm^2). The concentration of the nanocapsules is 4 μg mL^{-1}. Then the cells were stained with 10 μg mL^{-1} Hoechst 33342 and 10 μM DHE for another 30 min. After that, cells required illumination were irradiated by 660 nm light (35 mW/cm^2, 4 min, 8.4 J/cm^2). Cells were imaged using an OLYMPUSFV-1000 inverted confocal fluorescence microscope 60× oil mirror. The fluorescence of Hoechst 33342 was excited at 405 nm and collected on 420-480 nm. The fluorescence of DHE was excited at 488 nm and collected on 590-610 nm. The groups for hypoxia (oxygen $\leq$ 2%) constructed as instruction in *Cell incubation*, then the treatments were carried out: G1: Control; G2: FePtNBS@OVA; G3: FePtNBS@OVA+Light; G4: FePtNBS@OVA+DFOA+Light.

1.14 Intracellular Hydroxyl Radical Detection

The detection for hydroxyl radical was performed by using the similar procedure in *Intracellular superoxide anion free radical detection.* The concentration for HPF is 10 μM. The fluorescence of HPF was excited at 488 nm and collected on 500-550 nm.

1.15 Live-Dead Cell Staining

MCF-7 cells (approximately 2×10^5 cells/mL) were seeded in 35 mm diameter confocal dishes. After 24 h of culture, cells were treated with different administrations (Figure S4). The incubating time was 2 h. The concentration was 4 μg mL^{-1}, which was measured as the content of ovalbumin in the dosage form. The irradiation was elicited by a 660 nm LED, (35 mW/cm^2, 8 min, 16.8 J/cm^2). After different treatments, the treated drug was washed for three time with PBS. Then, co-staining of calcein AM and propidium iodide PI was performed after 30 min as instruction for the kit. The excitation wavelength was 488 nm, the emission wavelength of the green channel was 505-545 nm, and the emission wavelength of the red channel was 600-700 nm.

1.16 Apoptosis Experiment

MCF-7 cells (approximately 2×10^5 cells/mL) were seeded into 6-well plates. After 24 h of culture, cells were treated with different administrations (Figure S5). The concentration of nanocapsules is 4 μg mL^{-1}, which is measured as the content of ovalbumin in the dosage form. The irradiation was performed by a 660 nm LED light (35 mW/cm^2, 8 min, 16.8 J/cm^2) after 2 h incubation. After different treatments, the treated agents were washed for three times with PBS. Then, co-staining of Annexin-V and propidium iodide PI was performed after 30 min as instruction for the kit. After incubation, the cells were collected in flow tube, and finally the apoptosis effect in the cells was determined by analyzing 1×10^4 cells with FCM (Attune® NxT sonic focused flow cytometer).

1.17 In Vivo Fluorescence Imaging

All animal operations were preformed according to the institutional animal use and care regulations approved by Ethics Committee, Dalian University of Technology (Application No.: 2020-063). Female Balb/c mice, 6-8 weeks old, were obtained from the Experimental Animal Center of Dalian Medical University. 1×10^6 cells/mL of 4T1 cells were subcutaneously injected into the selected axillary site to establish a breast cancer tumor model of Balb/c mice. *In vivo* fluorescence imaging of mice was initiated as the tumor volume increased to approximately 200 mm^3. NBS (100 μL, 0.2 mg mL^{-1}) and FePtNBS@OVA (100 μL, 2 mg mL^{-1}) were injected intravenously into tumor-bearing mice and fluorescence imaging was observed at different post-injection times. In addition, mice treated with the same administration were dissected after 6 h, and tumor tissues and main organs (i.e., heart, liver, spleen, lung, and kidney) were imaged *ex vivo*. During the imaging process, the excitation wavelength was 635 nm and the collected emission wavelength was 680-720 nm.

1.18 *In Vivo* Therapeutic Efficacy

1×10^6 cells mL^{-1} of 4T1 cells were subcutaneously injected into the selected axillary site to establish a breast cancer tumor model of Balb/c mice. When the tumorous volume reached approximately 100 mm^3, the Balb/c mice in the 4T1 tumor model were divided into five groups and were treated with the following different treatments: G1: Control; G2: Fe@OVA; G3: FePtNBS+Light; G4: FePt@OVA; G5: FeNBS@OVA; G6: FeNBS@OVA+Light; G7: FePtNBS@OVA; G8: FePtNBS@OVA+Light. The administration dose for different nanocapsules is 1.5 mg mL^{-1}, which was calculated based on the ovalbumin capsules, and the injecting volume was 100 μL. The injected dose for G3 had the same mass ratio to FePtNBS@OVA. The tumor area was irradiated with a 660 nm xenon lamp with a power density of 150 mW/cm^2 for 20 min (180 J/cm^2), after post-injection for 6 h. Changes in tumor volume of all mice within 18 days were measured using vernier calipers after different treatments. The tumor volume = (width × width × length)/2. The survival rate was recorded in 28 days. Tumor tissues of the mice were harvested by hematoxylin-eosin (H&E) staining for histopathological analysis. In addition, the lungs of each group were collected. The metastatic facies were evaluated by hematoxylin and eosin (H&E) for analysis of lung metastasis.

2. Results

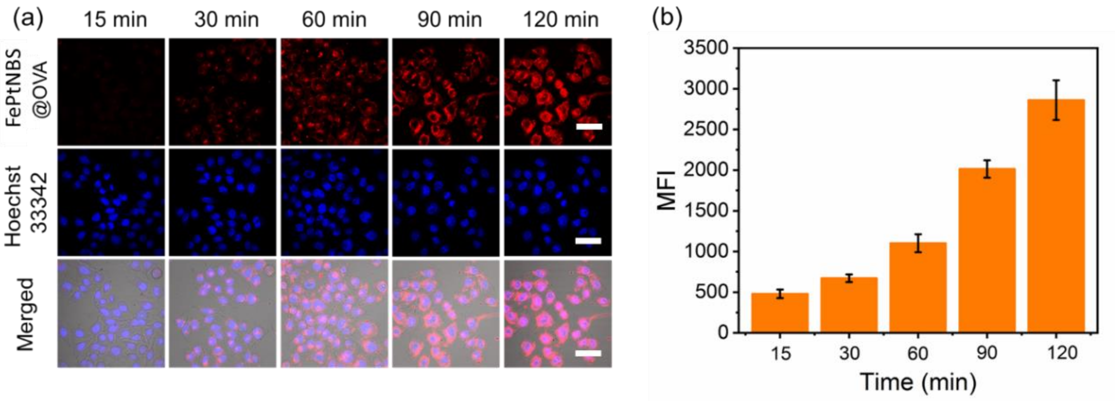

Figure S1. (a) Uptake of FePtNBS@OVA (2 μg mL^{-1}) in MCF-7 cells. Red fluorescence was excited at 635 nm and measured at 650-700 nm. The fluorescence of Hoechst 33342 was excited at 405 nm and measured at 420-480 nm. Scale bar: 40 μm. (b) Fluorescent intensity of FePtNBS@OVA obtained from (a).

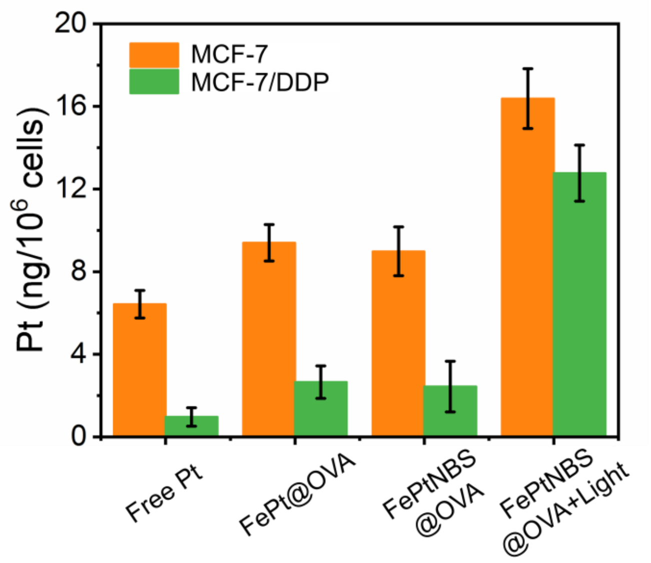

Figure S2. Intranuclear Pt content in MCF-7 and MCF-7/DDP cells with different treatments determined by ICP-MS. Data are given as the mean ± SD (n = 3). Cells in FePtNBS@OVA+Light were irradiated by a 660 nm LED light (35 mW/cm^2, 5 min, 10.5 J/cm^2).

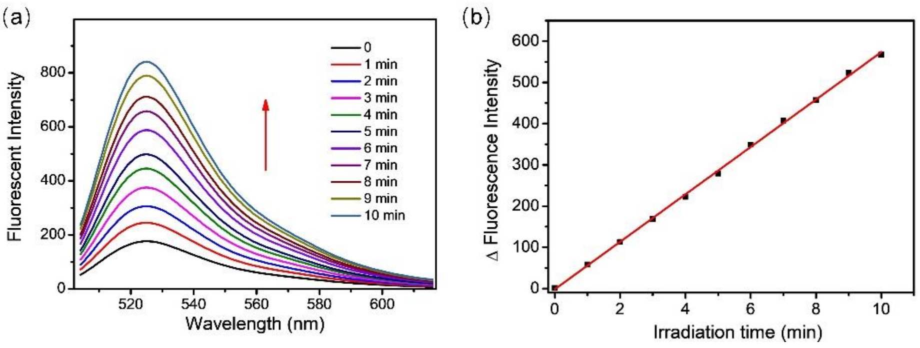

Figure S3. (a) Fluorescence spectra of DHR123 (10 μM) induced by the nanocapules (NBS 20 μM, the nanocapules were preincubated with trypsin at 37 °C for 30 h to release NBS) after irradiation for different time (660 nm, 15 mW/cm^2). (b) The change of emission intensity of DHR 123 (λ_{em}= 520 nm) was linearly related to irradiation time. Δ fluorescence intensity equals to fluorescence intensity in (a) minus fluorescence intensity of sole DHR 123, because light irradiation of DHR 123 can also significantly induce a fluorescence enhancement.

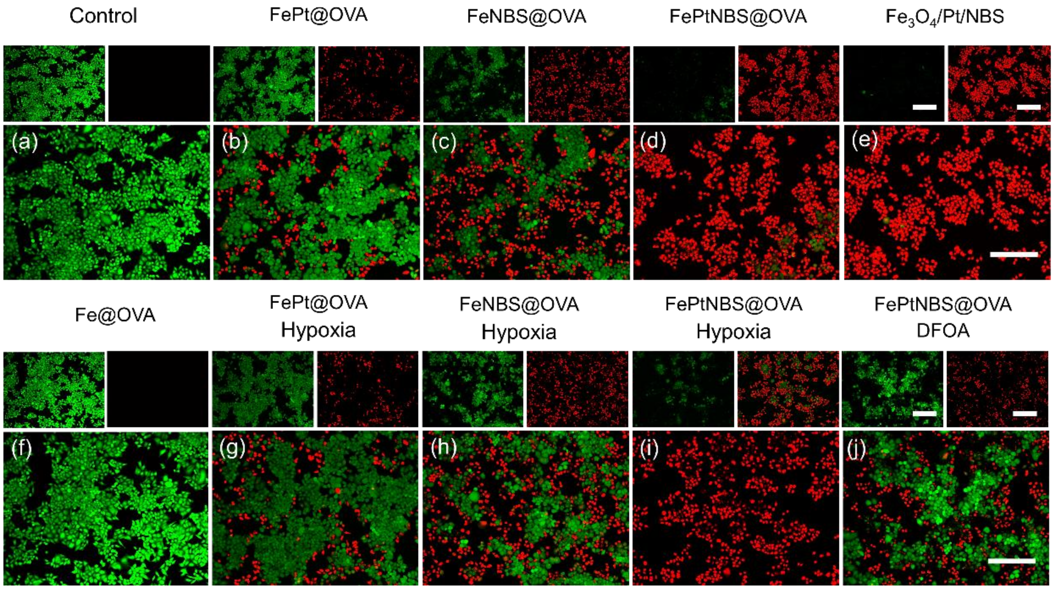

Figure S4. Live/Dead cell staining for MCF-7 cells with different treatments. The green channel is (λ_{ex} = 488 nm, λ_{em} = 500-530 nm) for the living cells and the red channel (λ_{ex} = 561 nm, λ_{em} = 560-600 nm) is for the dead cells. The concentration of nanocapsules was 4 μg mL^{-1}. The irradiation was elicited by a 660 nm LED, (35 mW/cm^2, 8 min, 16.8 J/cm^2). Scale bars: 300 μm.

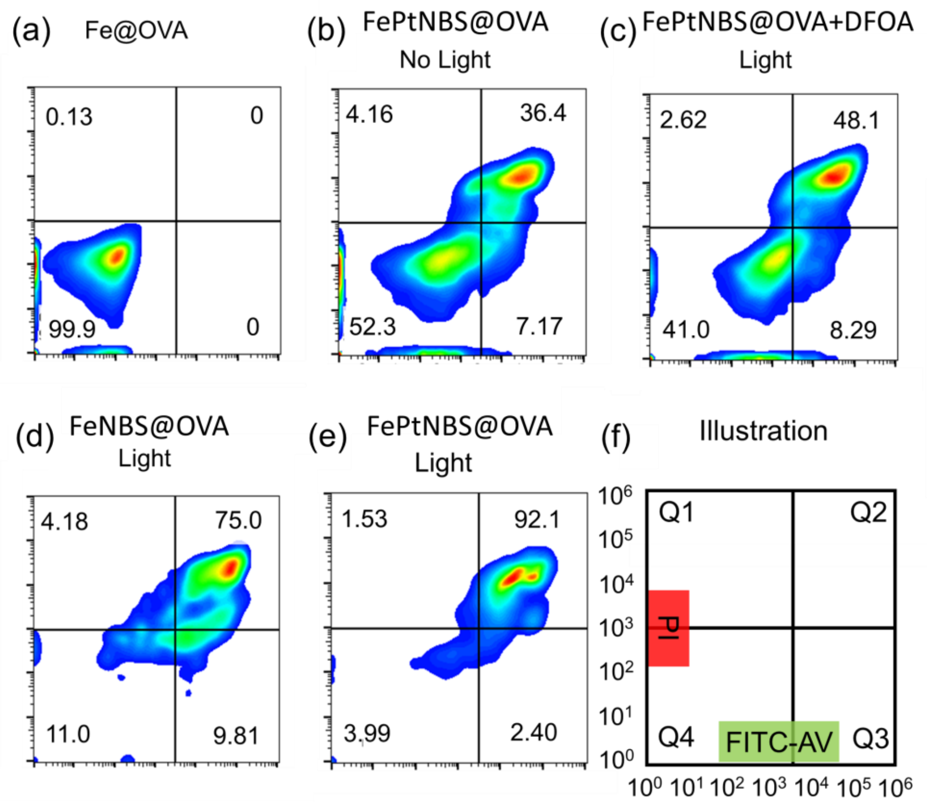

Figure S5. Flow cytometry analysis of apoptosis stage using AV-FITC and PI in MCF-7 with different treatments. The concentration of nanocapsules was 4 μg mL^{-1}. The light irradiation was performed by 660 nm LED light (35 mW/cm^2, 4 min, 8.4 J/cm^2) after 2 h incubation.

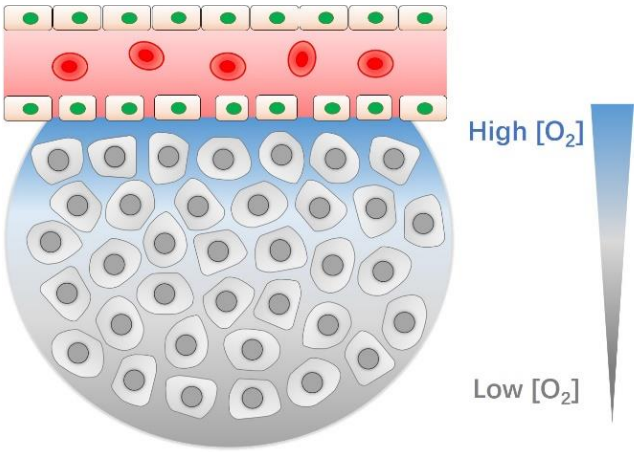

Figure S6. Schematic illustration of the pO_2 gradient from blood to hypoxic tumor center.

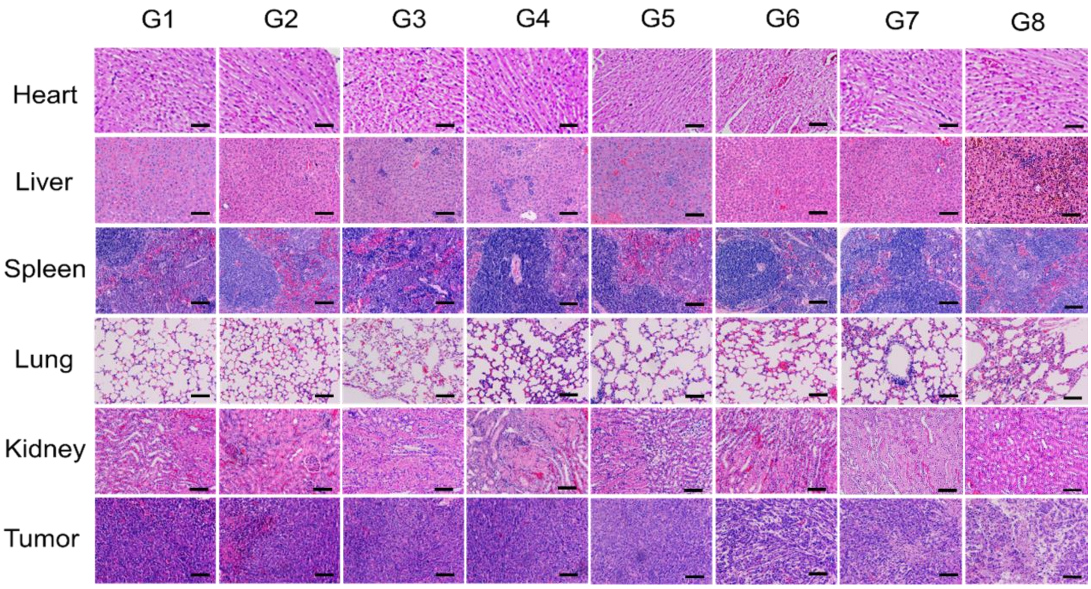

Figure S7. H&E staining of tumor tissues of different groups of mice. G1: Control; G2: Fe@OVA; G3: Fe/Pt/NBS+Light; G4: FePt@OVA; G5: FeNBS@OVA; G6: FeNBS@OVA+Light; G7: FePtNBS@OVA; G8: FePtNBS@OVA+Light.

References

[1] H. Schlaad, H. Kukula, J. Rudloff, I. Below, *Macromolecules* **2001**, *34*, 4302-4304.

[2] S. Verma, U. W. Sallum, H. Athar, L. Rosenblum, J. W. Foley, T. Hasan, *Photochemistry & Photobiology* **2009**, *85*.

[3] M. B. Bannwarth, S. W. Kazer, S. Ulrich, G. Glasser, D. Crespy, K. Landfester, *Angew Chem, Int Ed* **2013**, *52*, 10107-10111.

[4] M. B. Bannwarth, S. Ebert, M. Lauck, U. Ziener, S. Tomcin, G. Jakob, K. Münnemann, V. Mailänder, A. Musyanovych, K. Landfester, *Macromol Biosci* **2014**, *14*, 1205-1214.

[5] X. Li, V. Mak, Y. Zhou, C. Wang, E. Wong, R. Sharma, Y. Lu, A. Cheung, G. B. Mills, L. Cheung. *Nat. Commun.*, **2019**, 10, 30755611.

[6] S. Landskroner-Eiger, C. Qiu, P. Perrotta, M. Siragusa, M. Lee, V. Ulrich, A. Luciano, Z. Zhuang, F. Corti, M. Simons, R. Montgomery, D. Wu, J. Yu, W. Sessa. *P. Natl. Acad. Sci. USA* **2015**, *112*, 12812-12817.

[7] M. Pilato, E. Mejías-Pérez, M. Zonca, B. Perdiguero, C. Gómez, M. Trakala, J. Nieto, J. Nájera, C. Sorzano, C. Combadière, G. Pantaleo, L. Planelles, M. Esteban. *P. Natl. Acad. Sci. USA* **2015**, *112*, 1333-1342.

“大连理工大学生物与医学伦理委员会”申请审查表

Animal Experimental Ethical Inspection Form

of Dalian University of Technology

受理编号：2020-063

Application No.: 2020-063

以下内容由申请人填写 (Related information filled by applicant)			
申请人姓名 (Applicant)	孙文<br>Wen Sun		
所在院系 (Name of organization)	精细化工国家重点实验室<br>State Key Laboratory of Fine Chemicals, Dalian University of Technology		
申请人电话 (Tel)	13889596493	申请人电子邮件	sunwen@dlut.edu.cn

申请目的：（在相应的选择后画√）

Aim of Inspection: (Mark √ after the corresponding selection)

（1） 为发表学术论文进行伦理审查 (√)

For publication of academic papers

（2） 为申请科研项目进行伦理审查

For application of research projects

（3） 为其他目的进行伦理审查，请注明 ____________

Others

题目 (Title)：Synergistic Anticancer Therapy by Ovalbumin Encapsulation-Enabled Tandem ROS Generation

研究目的 (Aim of experiment)：

In the submitted manuscript, we report synergistic photodynamic therapy/chemotherapy with integrated tandem Fenton reactions mediated by ovalbumin encapsulation for improved in vivo anticancer therapy via an enhanced reactive oxygen species (ROS) generation mechanism. We

want to prove the anticancer effect of nanocapsules on tumor-bearing mice.<br>实验材料及研究方法（Materials and experimental methods）：<br>In the submitted manuscript, female Balb/c mice, 6-8 weeks old were used. All animal operations including the preparation of tumor-bearing mice, in vivo fluorescence imaging, tumor and organs isolation, and anticancer effect were conducted according to institutional animal use and care regulations approved by Dalian University of Technology.
负责人签字（表明负责人对以上情况知情）：<br>Signature of the applicant (indicating that the applicant is aware of the above situation) ： Wen Sun
以下内容由伦理委员会填写 (The following is to be completed by the ethics committee)
伦理委员会初步审查分类结果<br>Preliminary review of classification results by ethics committee<br>（1） 常规问题 Routine issues （√）<br>（2） 非常规问题 Unconventional issues<br>（3） 重大问题 Vital issues
伦理委员会审查意见 Attitude of the ethics committee<br>All animal experiments demonstrated in the submitted manuscript "Synergistic Anticancer Therapy by Ovalbumin EncapsulationEnabled Tandem ROS Generation" were preformed according to the institutional animal use and care regulations approved by Dalian University of Technology. All animal operations are in accordance with the ethical requirements of Dalian University of Technology.<br><br>签发人签字 Signature Hong Tang<br>大连理工大学生物与医学伦理委员会（盖章）<br>Ethics Committee, Dalian University of Technology (seal)<br>2020 年 07 月 12 日
